# Complex Clonal Diversity of* Staphylococcus aureus* Nasal Colonization among Community Personnel, Healthcare Workers, and Clinical Students in the Eastern Province, Saudi Arabia

**DOI:** 10.1155/2018/4208762

**Published:** 2018-12-18

**Authors:** Taghrid S. El-Mahdy, Mohamed H. Al-Agamy, Mohamed Emara, Assem Barakat, Richard V. Goering

**Affiliations:** ^1^Department of Microbiology and Immunology, Faculty of Pharmacy, Helwan University, Cairo, Egypt; ^2^Department of Microbiology, Faculty of Pharmacy, Ahram Canadian University, Cairo, Egypt; ^3^Department of Pharmaceutics, College of Pharmacy, King Saud University, Riyadh, Saudi Arabia; ^4^Microbiology and Immunology Department, Faculty of Pharmacy, Al-Azhar University, Nasr City, Cairo, Egypt; ^5^College of Science, King Saud University, Saudi Arabia; ^6^Department of Medical Microbiology and Immunology, School of Medicine, Creighton University, Omaha, NE, USA

## Abstract

Here, 210 healthy participants including community personnel (70), clinical students (68), and healthcare workers (HCWs) (72) from the eastern region of Saudi Arabia were studied. Sixty-three* Staphylococcus aureus* isolates were obtained from the nares of 37% of the community personnel and 26% of the clinical students and HCWs. Methicillin-resistant* S. aureus* (MRSA) was found in 16% (10 isolates) of the 63 isolates; six were from HCWs. Molecular characterization revealed high clonal diversity among the isolates, with 19 different* spa* types, 12 clonal complexes (CCs), and seven sequence types (STs) detected. The most common strain type was USA900, CC15, and t084, seen in 11 methicillin-susceptible* S. aureus* (MSSA) isolates. Moreover, three novel* spa* types in six isolates and one novel ST in two isolates were identified, most from HCWs. Interestingly, 29 isolates were* mec*A positive by PCR, whereas only 10 isolates were MRSA by disk diffusion (cefoxitin resistant). Of the 19 MSSA* mec*A-positive isolates, 16 were PBP2a negative, leaving three unique isolates from HCWs that were* mec*A and PBP2a positive yet cefoxitin susceptible. Our findings highlight the importance of phenotypically and genotypically characterizing* S. aureus* strains isolated from healthy communities to monitor the risk of possible cross-transmission to hospitalized patients. The identified strains showed a clonal lineage relationship with previously reported* S. aureus* and MRSA strains acquired from hospital settings.

## 1. Introduction

*Staphylococcus aureus* is among the most commonly isolated bacteria especially in hospitals. It causes different types of infections ranging from superficial lesions to life-threatening septicemia, endocarditis, and pneumonia [1]. Most* S. aureus* infections are caused by methicillin-sensitive strains (MSSA), although a worldwide increase in the number of infections and outbreaks caused by methicillin-resistant* S. aureus *(MRSA) are also evident [2]. Multiple body sites can be colonized by* S. aureus*, although in humans, the anterior nares are the most frequent carriage sites and are considered their native ecological niches. Although the nasal carriage of* S. aureus* does not indicate disease, it does increase the risk of acquiring staphylococcal infections. Consequently, it is used as an indicator to monitor the possibility of outbreaks and hospital-associated infections caused by this organism [3].

Healthcare workers (HCWs) including doctors, nurses, technicians, and diagnostic laboratory staff who have continuous hospital exposure represent an important reservoir for transmitting* S. aureus. *Similarly, clinical students during their internship are also potential sources for transferring the organism. Accordingly, screening the nasal carriage in these two cohorts is an important component of the control of* S. aureus *and MRSA in any healthcare facility [4, 5]. Although the community carriage rates of* S. aureus* are still low, they are rising rapidly in certain parts of the world [6], highlighting the importance of the rapid identification of carriers in order to appropriately control* S. aureus *infections.

The molecular characterization of* S. aureus* has become a routine tool for investigating circulating* S. aureus* clones, and the importance of detecting epidemic clones in hospitals as well as within the community has been well established. Pulsed-field gel electrophoresis (PFGE) has been considered the classical gold standard technique for molecular typing specifically for short-term epidemiology [7]. On the other hand, DNA sequence-based methods such as multilocus sequence typing (MLST) [8] and* S. aureus* protein A gene (*spa*)-typing [9] are replacing other molecular-typing techniques because of their ease of use in detecting international clones and exchanging results among laboratories via online databases [10].

The Kingdom of Saudi Arabia is the largest country in western Asia and the second largest in the Arab world. It occupies the bulk of the Arabian Peninsula. The city of Al-Ahsa is located in the eastern region of the kingdom. However, at present, the epidemiological and genetic characterization of* S. aureus* isolates, especially from healthy carriers, in regions of the Middle East including the Kingdom of Saudi Arabia remain insufficient. The aim of the current study was to determine the frequency of* S. aureus* and the predominant clones, especially including MRSA, carried in the anterior nares of an open population of community personnel, clinical students, and HCWs within the eastern region of the Kingdom of Saudi Arabia.

## 2. Materials and Methods

### 2.1. Subjects

A total of 210 healthy participants were included in the study: 70 were non-hospital personnel (adults in the community), 68 were clinical students, and 72 were HCWs. The non-hospital personnel were between 18 and 30 years old and had not been hospitalized, subjected to dialysis and/or surgery, implanted with a permanent indwelling catheter, or even administered an invasive medical device within the last year. Clinical students were pharmacy students who had clinical exposure during their internship in the eastern region of Saudi Arabia. All community personnel and clinical student participants were of Saudi nationality. The HCWs (doctors, nurses, and diagnostic laboratory staff) included different nationalities working in various clinical departments of six different hospitals in Al-Ahsa, Saudi Arabia. The participants who took part in the study were asked to sign a consent form.

### 2.2. Sampling Methods

Nasal samples were collected from the anterior nares of the three participant groups, HCWs, clinical students, and community personnel, using a transport swab moistened with sterile normal saline solution. Swabs were then placed in tubes containing Amies medium and transferred within 24 h for laboratory culture. The swabs were used to inoculate mannitol salt agar plates, which were then incubated at 37°C for 24 h.* S. aureus *was characterized by yellow colonies on MSA due to fermentation of mannitol and a positive coagulase and catalase tests. One representative isolate of* S. aureus* from each plate was subcultured, screened for coagulase (Staphaurex, Remel, Lenexa, KS, USA), and preserved at -80°C.

### 2.3. Antimicrobial Susceptibility Testing

*S. aureus* isolates were screened for their susceptibility to six antibiotics (erythromycin, 15 *μ*g; ampicillin, 10 *μ*g; cefoxitin, 30 *μ*g; amoxicillin/clavulanic acid, 20/10 *μ*g; linezolid, 30 *μ*g; and clindamycin, 2 *μ*g) using the disk diffusion method (Oxoid Ltd, Basingstoke, UK) following the Clinical Laboratory Standards Institute (CLSI) guidelines [11]. Disk diffusion analysis of cefoxitin resistance to detect MRSA was performed according to CLSI recommendations.

### 2.4. Expression of the mecA Gene (i.e., PBP2a Production)

Penicillin Binding Protein 2a (PBP2a) was assessed by either rapid latex agglutination (Oxoid PBP2a Latex Agglutination Test, Oxoid Microbiology Products, Basingstoke, Hampshire, UK) or rapid immunochromatographic qualitative assay (Alere PBP2a, Alere™, Waltham, MA, USA).

### 2.5. Molecular Characterization

#### 2.5.1. PFGE Analysis

For each isolate, chromosomal DNA was extracted, digested by* Sma*I restriction endonuclease in agarose plugs, and analyzed as previously described [12]. Isolates with a PFGE profile similarity of 80% or higher (Dice coefficient, UPGMA clustering) were considered to be related as per Tenover et al. [7].

#### 2.5.2. PCR Amplification of the* mecA* Gene

Genomic DNA was extracted and utilized for PCR amplification of the* mecA* gene as described by Fey et al. [13].

#### 2.5.3. Staphylococcal Cassette Chromosome* mec* (SCC*mec*) Typing

SCC*mec* typing was performed on* mec*A-positive isolates using previously described multiplex PCR protocols [14, 15].

#### 2.5.4. *spa* Typing

PCR for* spa* typing was performed using the primers and thermal cycling conditions of the European Network of Laboratories for Sequenced Based Typing of Microbial Pathogens (SeqNet [http://www.seqnet.org]). The* spa* typing plug-in tool of BioNumerics v7.5 was used for analysis of* spa* sequences and assignment of* spa* types, which were confirmed via the freely available Ridom Spa Server (http://spa.ridom.de/index.shtml).

#### 2.5.5. MLST

MLST was performed according to the protocol on the* S. aureus* MLST website (http://saureus.mlst.net/misc/info.asp).

## 3. Results

### 3.1. S. aureus Nasal Carriage and Antimicrobial Susceptibility Testing

*S. aureus* was isolated from 26 (37%) of the non-hospital community personnel, 18 (26%) of the clinical students, and 19 (26%) HCWs, for a total of 63 isolates. As shown in Table 1, methicillin resistance was found in 16% of the* S. aureus* isolates (one from the community, six from HCWs, and three from clinical students). Multiresistant* S. aureus* (resistant to three antibiotics) was isolated from two HCWs (one was MRSA), one community personnel and one clinical student (both isolates were MRSA). Resistance to ampicillin was the most prevalent in all population groups (90% of isolates). No resistance to linezolid was detected, whereas amoxicillin/clavulanic acid and clindamycin remained potent antimicrobials, with 92% and 98% potency, respectively. Five isolates were fully susceptible, three isolated from clinical students and two from community personnel, whereas there were no fully susceptible isolates from HCWs.

### 3.2. S. aureus Molecular Characterization and Typing

**Figure 1:**
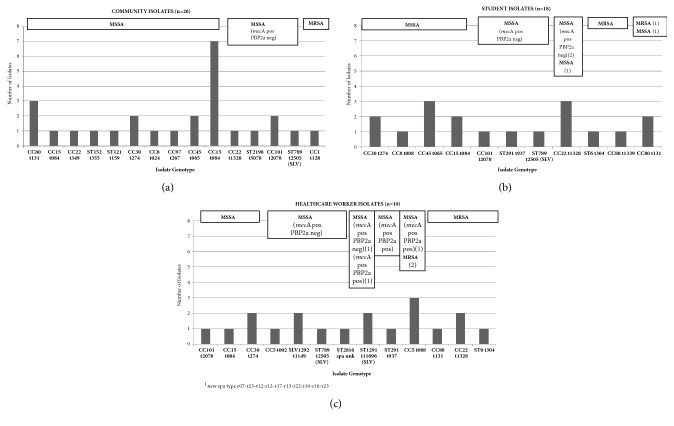
Isolates genotypes of the three cohorts.

The* S. aureus* strain types recovered from the different participant groups are summarized in Table 2 and Figure 1. Isolates were initially characterized by PFGE (see supplemental file (available here)) and, where possible, assigned to known* S. aureus* strain types. Uncertain assigned isolates to known international PFGE types were further characterized by* spa* typing, from which the MLST clonal complex (CC) or sequence type (ST) was either inferred or specifically determined.

The 26 community isolates included 14 different strain types (Figure 1(a); Table 2), which in some cases were related to well-known epidemic MRSA types (e.g., CC80 t131; CC8 t024); however, only one was methicillin resistant (CC1 t128). The overwhelming majority of isolates (20 of 26, 77%) were “true” MSSA, known by cefoxitin susceptibility in disk diffusion test, and the most common strain type was CC15 t084 (8 isolates). Five isolates representing four different strain types were phenotypically methicillin susceptible but carried a presumably inactive* mec*A gene, as evidenced by the susceptible cefoxitin disk diffusion inhibition zone size and the absence of a PBP2a product.

The 18 clinical student isolates (Figure 1(b); Table 2) included 11 different strain types, which in most cases were the same as those isolated from the community personnel. However, three isolates with three different genetic backgrounds were methicillin resistant (ST6 t304, CC88 t1339, and CC80 t131). In addition, five isolates representing four different strain types (three of which were also seen in the community personnel group) exhibited phenotypic methicillin susceptibility, despite carriage of a presumably inactive* mec*A gene.

The 19 isolates from HCWs (Figure 1(c); Table 2) comprised 13 different strain types. This group had the highest number of “true” MRSA (known by cefoxitin resistance in disk diffusion analysis), 32%: six isolates representing four different strain types and exhibiting greater genetic variability than the two other groups. Six isolates representing four different strain types were phenotypically methicillin susceptible with an inactive* mec*A gene; one of these strain types was seen in another group (ST789 SLV-t2505). However, the HCW group was unique in yielding three isolates representing three different strain types that were phenotypically methicillin susceptible but* mec*A positive and PBP2a positive.

The most common genotype was USA900, CC15, and t084, which was detected in 11 MSSA isolates: eight from community personnel, two from clinical students, and one from HCWs. The next most frequent types (six isolates each) were USA200, CC30, t274; EU ST80, CC80, and t131; and EMRSA-15, CC22, and t1328 (Table 2). One-third of the* S. aureus* isolates (21 of 63) did not correspond to any known PFGE strain type and were thus assigned to 12 different PFGE groups (A to L). SCC*mec* typing was performed for all 29* mec*A-positive isolates, with SCC*mec*IV being the most common, and represented by 13 isolates. Eight of the 10 MRSA (cefoxitin resistant) isolates were SCC*mec* type IV, and the remaining two were SCC*mec* II (Table 2). Three novel* spa* types were found in six isolates as well as one novel ST (SLV “single locus variant” 1292) in two isolates; six of the eight isolates were from HCWs.

## 4. Discussion

The striking finding in the present study was the higher than expected nasal colonization rate (37%, 26 of 70) of* S. aureus* among the community personnel compared to clinical students (26%) and HCWs (26%), all of whom had clinical exposure. On the other hand, the rate of MRSA was the highest among HCWs (six isolates out of 19, 32%), whereas it was the lowest among community personnel (one isolate out of 26, 4%). Two studies reported the characterization of* S. aureus* from nasally colonized HCWs and medical students in Saudi Arabia [[16, 17], respectively]. In the first study, 40% (80 of 200) of HCWs (primarily nurses) were* S. aureus *carriers, with 36 of 80 isolates (45%) MRSA. In the second study, 25% (38 of 150) of students were nasal carriers of* S. aureus*, 10 of them (all from interns who underwent clinical training) being MRSA carriers as determined by detection of the* mec*A gene. These data are similar to ours, in which most MRSA isolates (nine of 10 isolates) were found among HCWs and clinical students, indicating that hospital exposure may lead to acquiring MRSA strains. Furthermore, Zakai [16] used the CLSI 2010 guidelines when reporting that only two of 10* mec*A-positive isolates were oxacillin resistant by disk diffusion, whereas we identified MRSA as cefoxitin resistant by disk diffusion following the CLSI 2015 recommendations.

A study by Laman et al. has reported a nasal colonization of* S. aureus* in 44 (17.1%) of 257 samples with four isolates being methicillin resistant [18]. In another recent study, Heckel and coworkers reported an overall MRSA carriage rate of 4.1% in a German specialist palliative care setting and the prevalence rate of MRSA in PCU patients was higher than in general acute hospital populations [19]. Moreover, in a study from Taiwan [20], 26% of high school students were found to carry* S. aureus* in their noses, 14% of which were MRSA. This finding differs from our results for community personnel, among whom there was a 37% (26 of 70) rate of nasal carriage of* S. aureus* but with only one MRSA isolate (4%).

The current study revealed a high clonal diversity among* S. aureus* isolates from the three cohorts of healthy nonpatient individuals (community personnel, clinical students, and HCWs). The highest degrees of diversity and genetic variability were seen in the HCW group. Nineteen different* spa* types, 12 CCs, and seven STs were detected (Table 2), confirming the large genetic variability of isolates found in clinical settings. This is in contrast to a previous report noting a greater diversity of MRSA strains acquired from the community in comparison to those from a hospital setting [10]. In a study from Korea by Kang and colleagues, they reported a concordance rate of 94.2% between colonizing and clinical isolates by methicillin susceptibility with ST72-SCCmec type IV being the most predominant clone [21].

This study underscores the potential for the international dissemination of known epidemic* S. aureus* strain types to the eastern region of Saudi Arabia because two-thirds of the isolates (42 of 63) corresponded to well-known PFGE types (see supplemental file). It should be noted that the majority of these known types (32 of 42) were from community personnel and clinical students, who were all of Saudi nationality. Although the PFGE characterization of* S. aureus* isolates from Saudi Arabia has been performed previously [22–24], no comparison of relatedness with known international strains was reported (e.g., for MRSA isolates), making it difficult to compare that data with our findings. On the other hand, an 18-year-old study by Van Belkum et al. [25] found that the overwhelming majority of Saudi Arabian MRSA strains (93%) at that time clustered into one predominant type with no relationship to any known epidemic clone. Okoye et al. reported a potential reduction (3-fold reduction) in the prevalence of MRSA nasal colonization in children admitted to Driscoll Children's Hospital. They also reported that, out of 360 children, 21% were colonized with* S. aureus* and 14% of those isolates were MRSA [26].

In the present study, 17% of isolates (11 of 63) were identified as the CC15, t084 clone, and all were MSSA. This is in accordance with a previous study from Ghana [27], where 19% (57 of 308) of their isolates were CC15, 37 were t084, and all were MSSA. The CC15 t084 clone was also detected in 27% of MSSA isolates in a Swedish study [28]. It is worth mentioning that, in both of these previous studies, the MSSA isolates were clinical isolates in contrast to our study where all isolates were from healthy personnel, suggesting the cross-dissemination of clones between community and hospital settings. In Saudi Arabia, CC15 (t084, t085) was also detected at a high incidence (27%) in MSSA and MRSA clinical isolates from the AL-Qassim district, which is located in the center of Saudi Arabia, indicating the successful movement and persistence of this clone [29]. The other less commonly identified clonal complexes in our study (CC30, CC80, CC22, and CC45) have also been reported in clinical MRSA isolates from Riyadh (in middle region and the capital city of Saudi Arabia), whereas CC22 was the most prevalent clone (28%) [30]. In the present study, 13 of the 29 (45%)* mec*A-positive isolates were SCC*mec*IV. This result is in contrast to data from the city of Makkah (in the western region of Saudi Arabia), where SCC*mec*III was the most prevalent (47%) followed by type IV (29%) [31]. However, another study from Saudi Arabia reported that SCC*mec*IV represented 75% (80 of 107) of isolates [30].

In another study done in Riyadh, Saudi Arabia;,17 clonal complexes were identified in* S. aureus* isolated from medical students, namely, CC15-MSSA, CC1-MSSA-SCCfus, CC8-MSSA, CC22-MSSA, CC25-MSSA, CC101-MSSA, CC5-MSSA, CC6-MSSA, CC30-MSSA, CC45-MSSA, CC96-MSSA, CC188-MSSA, CC398-MSSA, CC942-MSSA/PVL+, CC1290-MSSA, ST2482-MSSA, and CC80-MRSA-IV/PVL+ [32].

We found 19 MSSA isolates (30%) that carried the* mec*A gene but were still cefoxitin susceptible; 16 of these were PBP2a negative, leaving three unique MSSA isolates from HCWs that were positive for both* mec*A and PBP2a yet cefoxitin susceptible. These results illustrate the dynamic interrelationship between the presence or the absence of SCC*mec* and its expression in* S. aureus* strains. Previous reports have shown that MRSA and MSSA may contain similar genetic backgrounds, with the intermittent acquisition of SCC*mec* in MSSA populations [e.g., [33]]. In addition, the spontaneous excision of SCC*mec*, which encodes the protein responsible for methicillin resistance, may convert MRSA strains to MSSA, which could aid in the treatment of serious infections with resistant strains [34]. The latex agglutination test for PBP2a may show false positive reactions with MSSA [35], which although infrequent may explain our three unique isolates. However, the presence of* mecA* and PBP2a in MSSA strains deserves further investigation. With regard to susceptibility testing, these data suggest that the cefoxitin disk diffusion test is the most reliable means of identifying functionally active MRSA even though it takes longer than the latex agglutination test for PBP2a and PCR detection of* mec*A.

## 5. Conclusion

The diversity and complexity of the* S. aureus* strains isolated in our study, which were more related to previously known epidemic strains, underscore the need for the routine screening of healthy carriers to prevent infections caused by cross transmission. This study also highlights the importance of both the phenotypic and genotypic characterization of MRSA to better identify the carriers of resistant strains, especially among healthcare staff and clinical students who may serve as a reservoir of MRSA.

## Figures and Tables

**Table 1:** *S. aureus* carrier demographic data and isolate resistance patterns.

**Cohort**	**Isolate code**	**Age (years)**	**Sex**	**Relatives in healthcare facility**	**Hospitalization during last year**	**Use of Ab in the last year**	**Resistance pattern** ^**a**^
**Non-hospital personnel (community)**	2C	20	F	N	N	Y	AMP, E
	3C	21	F	N	N	Y	AMP
	6C	21	F	Y	N	N	AMP
	7C	20	F	N	N	Y	AMP
	10C	19	F	N	N	Y	AMP
	13C	21	F	Y	N	Y	AMP
	14C	21	F	Y	N	N	AMP, AMC
	15C	21	F	N	N	N	AMP
	17C	19	F	N	N	Y	AMP
	18C	19	F	N	N	N	-^b^
	19C	22	F	N	N	Y	AMP, AMC
	22C	22	M	N	N	Y	AMP
	24C	21	M	N	N	Y	AMP
	25C	21	M	Y	N	N	AMP
	27C	22	M	N	N	Y	AMP
	29C	22	M	Y	N	N	AMP
	35C	21	M	N	N	N	AMP
	41C	23	F	N	N	N	AMP
	45C	20	F	N	N	N	AMP
	50C	24	F	N	N	Y	-^b^
	54C	22	F	N	N	Y	AMP, AMC, FOX
	56C	22	F	N	N	N	AMP
	57C	22	F	Y	N	N	AMP
	63C	20	F	N	N	N	AMP
	67C	19	F	N	N	N	AMP
	69C	21	F	N	N	N	AMP

**Healthcare workers**	2W	26	F	Y	N	N	AMP, FOX
	12W	27	M	N	N	N	AMP
	15W	26	F	N	N	Y	AMP
	17W	32	M	N	N	Y	E
	23W	24	M	Y	N	N	AMP
	28W	27	F	N	Y	N	AMP, FOX
	30W	25	F	N	N	N	AMP, E
	31W	26	M	N	N	N	AMP
	33W	25	F	N	N	Y	AMP
	38W	23	M	N	N	N	AMP
	49W	23	M	Y	N	N	AMP
	50W	24	M	N	N	Y	AMP
	55W	40	F	Y	Y	N	AMP, FOX
	57W	59	M	N	N	N	AMP, E, DA
	58W	53	M	Y	N	N	AMP, FOX
	63W	31	M	N	Y	N	AMP, FOX
	68W	42	M	Y	N	N	AMP, AMC, FOX
	70W	34	M	N	N	N	AMP
	73W	23	M	N	N	N	AMP

**Clinical students**	3S	23	F	Y	Y	Y	AMP
	5S	22	F	Y	N	Y	AMP
	10S	22	F	N	Y	Y	-^b^
	11S	22	F	N	N	N	-^b^
	16S	21	F	N	N	N	AMP
	17S	23	F	Y	Y	Y	AMP, FOX
	19S	22	F	Y	Y	Y	AMP
	24S	23	F	N	N	Y	AMP, E
	26S	22	F	N	N	Y	AMP
	30S	22	F	Y	N	Y	AMP
	35S	23	M	Y	N	N	AMP
	37S	23	M	Y	N	Y	AMP
	38S	23	M	N	N	Y	AMP
	39S	22	M	N	N	N	-^b^
	45S	25	M	N	N	N	AMP
	48S	22	M	N	N	Y	AMP
	58S	21	F	N	Y	Y	AMP, AMC, FOX
	68S	22	M	Y	N	Y	AMP, FOX

M: male; F: female.

Y: yes; N: no.

^a^resistance determined by disk diffusion method, interpreted according to CLSI guidelines [11].

E: erythromycin, 15 *μ*g; AMP: ampicillin, 10 *μ*g, FOX: cefoxitin, 30 *μ*g; AMC: amoxicillin/clavulanic acid, 20/10 *μ*g; LZD: linezolid, 30 *μ*g; DA: clindamycin, 2 *μ*g.

^b^no resistance detected.

**Table 2:** Molecular characteristics of *S. aureus* isolates.

**Genotype (PFGE** ^**a**^ **, MLST, *spa*) / no. of isolates (from C, S, W)**	**No. of MSSA **	**No. of MRSA** ^**b**^ ** (SCC*mec*) / isolate codes**
USA900, CC15, t084 / 11 (8, 2, 1)	11	0

EU ST80, CC80, t131 / 6 (3, 2, 1)	4	2 (IV) / 58S, 68W

EMRSA-15, CC22, t1328 / 6 (1, 3, 2)	4 (3 *mec*A pos with SCC*mec* IV but PBP2a neg)	2 (IV) / 28W, 63W

USA200, CC30, t274 / 6 (2, 2, 2)	6	0

USA600, CC45, t065 / 5 (2, 3, 0)	5	0

USA800, CC5, t688 / 3 (0, 0, 3)	1 (*mec*A pos with SCC*mec* IV and PBP2a pos)	2 (IV) / 2W, 55W

USA100/800, CC5, t002 / 1 (0, 0, 1)	1 (*mec*A pos with SCC*mec* II but PBP2a neg)	0

USA300/500, CC8, t008 / 1 (0, 1, 0)	1	0

USA300/500, CC8, t024 / 1 (1, 0, 0)	1	0

USA500, CC97, t267 / 1 (1, 0, 0)	1	0

USA400, CC1, t128 / 1 (1, 0, 0)	0	1 (IVa) / 54C

PFGE A, ST1291, unknown *spa*^c^ / 2 (0, 0, 2)	2 (1 *mec*A pos with SCC*mec* V and PBP2a pos; 1 *mec*A pos with SCC*mec *V but PBP2a neg)	0

PFGE B, ST2196, t5078 / 1 (1, 0, 0)	1 (*mec*A pos with SCC*mec* I but PBP2a neg)	0

PFGE C, CC101, t5078 / 4 (2, 1, 1)	4 (3 *mec*A pos with SCC*mec* I but PBP2a neg)	0

PFGE D, ST291, t937 / 2 (0, 1, 1)	2 (1 *mec*A pos with SCC*mec* I and PBP2a pos; 1 *mec*A pos with SCC*mec *I but PBP2a neg)	0

PFGE E, SLV1292, t1149 / 2 (0, 0, 2)	2 (*mec*A pos with SCC*mec* V but PBP2a neg)	0

PFGE F, ST6, t304 / 2 (0, 1, 1)	0	2 (II) / 17S, 58W

PFGE G, ST789, unknown *spa*^d^ / 3 (1, 1, 1)	3 (*mec*A pos with SCC*mec* I but PBP2a neg)	0

PFGE H, CC22, t349 / 1 (1, 0, 0)	1	0

PFGE I, ST152, t355 / 1 (1, 0, 0)	1	0

PFGE J, ST2816, unknown *spa*^e^ / 1 (0, 0, 1)	1 (*mec*A pos with SCC*mec* IV but PBP2a neg)	0

PFGE K, CC88, t1339 / 1 (0, 1, 0)	0	1 (IV) / 68S

PFGE L, CC121, t159 / 1 (1, 0, 0)	1	0

^a^PFGE designations either indicate a relationship to known strain types or, where unknown, an arbitrarily assigned alphabetical designation

^b^MRSA as determined by resistance to cefoxitin

^c^unknown *spa* type (SLV of t11096) 04-12-17-20-17-12-17-17-16

^d^unknown *spa* type (SLV of t2505) 07-23-21-17-13-323-23-02-12-23

^e^unknown *spa* type 07-23-12-12-17-13-22-16-16-23

C: community personnel; W: health care workers; S: clinical students

pos: positive; neg: negative.

## Data Availability

The data used to support the findings of this study are included within the article.

## Supplementary Materials

Supplementary Materials PFGE dendogram for molecular typing of the isolates.
