# Supplementary figures and images for: Complex Clonal Diversity of Staphylococcus aureus Nasal Colonization among Community Personnel, Healthcare Workers, and Clinical Students in the Eastern Province, Saudi Arabia
Source: Biomed Res Int. 2018 Dec 18;2018:4208762. doi: 10.1155/2018/4208762 (PMC6312594; doi:10.1155/2018/4208762)

PFGE

PFGE

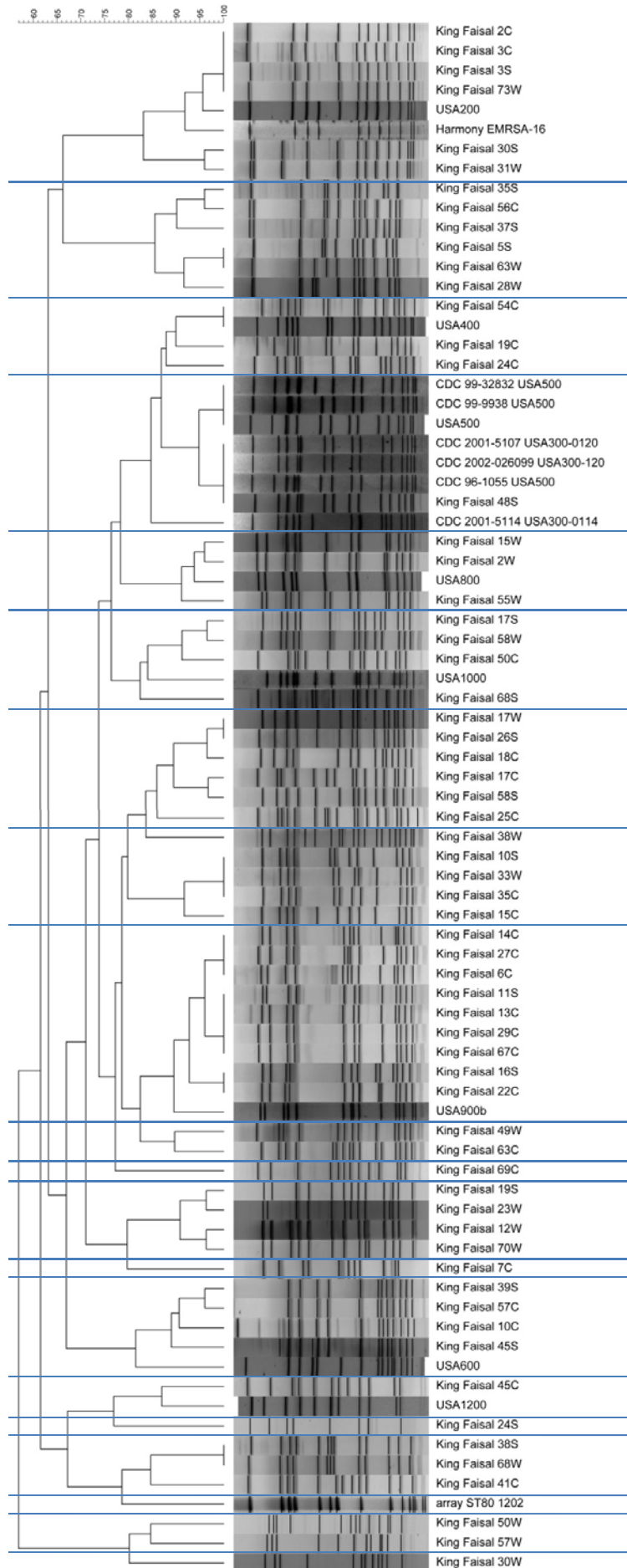

Supplement: Supplementary Materials — PFGE dendogram for molecular typing of the isolates. [file 4208762.f1.pdf]
